# Supplementary material for: Adolescent–Parent Agreement on Callous–Unemotional Traits in Adolescents with Attention-Deficit/Hyperactivity Disorder
Source: Int J Environ Res Public Health. 2020 May 30;17(11):3888. doi: 10.3390/ijerph17113888 (PMC7312037; doi:10.3390/ijerph17113888)
Supplement: Supplementary file 1 [file ijerph-17-03888-s001.pdf]

International Journal of
Environmental Research and Public Health

MDPI

Table 1. Adolescent–parent agreement ratings on every items of the Chinese version of the Inventory of Callous and Unemotional Traits.

	Adolescent-Report Mean (SD)	Parent-Report Mean (SD)	Paired *t*	*P*	ICC
1. Expressing feelings openly	1.3 (0.9)	1.5 (0.9)	−4.332	<0.001	0.207
2. Thinking different about "right" and "wrong" from others	1.3 (0.9)	1.3 (0.9)	1.238	0.216	0.158
3. Caring about doing well at school or work	1.7 (1.0)	1.6 (0.9)	1.228	0.220	0.272
4. Not caring about hurting others to get somethings	0.4 (0.8)	0.5 (0.8)	−2.525	0.012	0.258
5. Feeling bad or guilty when doing something wrong	1.8 (1.0)	1.7 (0.9)	1.861	0.063	0.136
6. Not showing emotions to others	1.1 (0.9)	1.0 (0.9)	3.274	0.001	0.104
7. Not caring about being on time.	0.6 (0.9)	1.0 (1.0)	−7.280	<0.001	0.262
8. Being concerned about the feelings of others	1.8 (1.0)	1.5 (0.9)	5.848	<0.001	0.201
9. Not caring about getting into trouble	0.6 (0.9)	0.9 (0.9)	−6.230	<0.001	0.232
10. Not being controlled by feeling	1.2 (0.9)	0.9 (0.8)	6.786	<0.001	0.174
11. Not caring about doing things well	0.6 (0.8)	1.0 (0.9)	−8.418	<0.001	0.195
12. Being cold and uncaring to others	0.6 (0.9)	0.6 (0.8)	0.580	0.562	0.270
13. Easily admitted to being wrong.	1.3 (1.0)	1.1 (0.9)	4.847	<0.001	0.241
14. Being easy for others to tell my feeling	1.3 (0.9)	1.7 (0.9)	−8.981	<0.001	0.148
15. Always trying best	1.8 (0.9)	1.3 (0.9)	10.713	<0.001	0.270
16. Apologizing to hurting persons.	2.0 (0.9)	1.7 (0.9)	6.686	<0.001	0.308
17. Trying not to hurt others' feeling	1.9 (1.0)	1.5 (0.9)	8.591	<0.001	0.321
18. Not feeling remorseful when doing something wrong	0.5 (0.8)	0.7 (0.8)	−4.837	<0.001	0.214
19. Being expressive and emotional	1.5 (1.0)	1.5 (1.0)	−0.609	0.543	0.193
20. Not like to put the time into doing things well	0.7 (0.9)	1.1 (1.0)	−9.162	<0.001	0.227
21. Not thinking the feelings of others important	0.5 (0.8)	1.1 (1.0)	−11.959	<0.001	0.241
22. Hiding feelings from others	1.2 (1.0)	1.0 (0.9)	2.449	0.015	0.179
23. Working hard on everything	1.8 (0.9)	1.4 (0.9)	8.852	<0.001	0.310
24. Doing things to make others feel good	1.6 (0.9)	1.2 (0.8)	7.541	<0.001	0.254

ICC: Intraclass correlation; SD: standard deviation.
